# A novel immuno-device based on the specific binding of AuNP-supported CTAB with biotinylated antibody of hyaluronic acid toward an early-stage recognition of a biomarker: a bioanalytical assay in real samples using disposal biosensor technology†

**DOI:** 10.1039/d2ra04984h

**Published:** 2022-10-05

**Authors:** Ahmad Mobed, Fereshteh Kohansal, Sanam Dolati, Mohammad Hasanzadeh

**Affiliations:** Infectious and Tropical Diseases Research Center, Tabriz University of Medical Sciences Tabriz Iran; Physical Medicine and Rehabilitation Research Center, Aging Research Institute, Tabriz University of Medical Sciences Tabriz Iran sanam.dolati@gmail.com; Pharmaceutical Analysis Research Center, Tabriz University of Medical Sciences Tabriz Iran hasanzadehm@tbzmed.ac.ir

## Abstract

Hyaluronic Acid (HA) is a non-sulfated glycosaminoglycan, which is a potential biomarker that could be evaluated in the diagnosis of some cancers. For the first time, a novel label-free electrochemical immunosensor was developed based on modified ITO-PET (indium tin oxide-polyethylene terephthalate) electrodes for the sensitive recognition of hyaluronic acid (HA) in real samples. A disposable ITO-coated PET electrode was modified with gold nanoparticles (AuNPs) to construct a suitable substrate for the efficient immobilization of biotinylated antibodies of HA. Importantly, the encapsulation of biotinylated antibody of HA in KCC1-NH-CS_2_ was performed successfully, which was another innovative part of this bio-device construction. For determining the immobilization steps and optimization of the biosensor, electrochemical impedance spectroscopy (EIS) and cyclic voltammetry (CV) techniques were used. Furthermore, the morphological characterization of each ITO electrode surface was performed by field emission scanning electron microscopy (FESEM). Specific binding of gold nanoparticles supported CTAB to ITO-PET and its bioconjugation with the biotinylated antibody of HA was studied using the electroanalysis of the sensor performance. For the better performance of the antibody to generate an immunocomplex with HA (antigen), its encapsulation was performed, which led to the excellent behavior of the immunosensor. The proposed HA immunosensor indicated excellent reproducibility, high selectivity, and long-term stability. The HA electrochemical immunosensor performed perfectly with a wide determination range (0.078 to 160 ng mL^−1^) and a low limit of quantification (0.078 ng mL^−1^) in human plasma samples. It is recommended that the designed biosensor can be used as a diagnostic tool in clinical bioassays in the near future.

## Abstract 2

Hyaluronic Acid (HA) is a non-sulfated glycosaminoglycan, which is a potential biomarker that could be evaluated in the diagnosis of some cancers.

## Graphical abstract

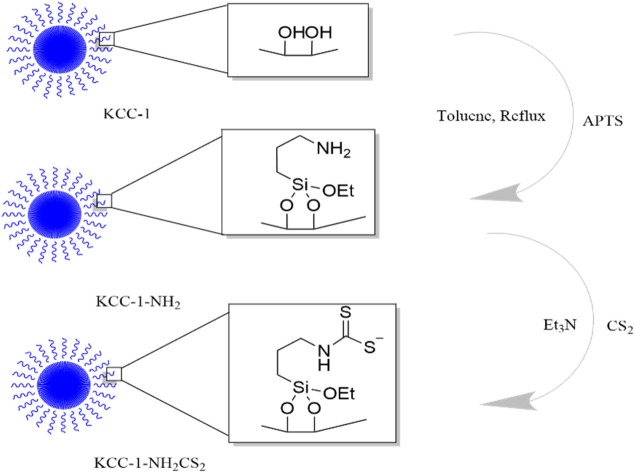

## 1. Introduction

Hyaluronic acid (HA) is an unbranched glycosaminoglycan consisting of the repeating disaccharide units of *N*-acetyl-d-glucosamine and d-glucuronic acid.^1^ As a major glycosaminoglycan in the extracellular matrix (ECM), HA can modulate the tumor microenvironment to promote the malignant phenotype, and it has been described by its effect on numerous signaling pathways involved in cancer and through the upregulation of collagen-degrading enzyme expression.^2^ Most commonly, hyaluronan was synthesized as a high-molecular-mass polymer, with an average molecular mass of approximately 1000–8000 kDa.^3^ The turnover in the bloodstream is normally in the range of 0.3–1.0 pg min- per kg body weight.^4^ Hyaluronan is synthesized by hyaluronan synthase on the inner surface of the cell membrane and translocated into the extracellular space along with the elongation of polymer chains.^5^ This is a unique method of synthesis that differs from other GAG synthesized techniques in the intracellular space. Hyaluronic acid is also the only GAG that does not bind to core proteins and undergo post-synthetic modifications.^6^ Long hyaluronic acid polymers have the ability to bind to large amounts of water. The hygroscopic and viscoelastic properties of HA make it a perfect component of the vitreous humor, synovial fluid and dermis. Hyaluronic acid in its very long polymer native form is known as high molecular weight (HMW) hyaluronic acid.^7^ However, under certain conditions, it can be broken down into smaller fragments called low molecular weight HA (LMWHA). Hyaluronic acid turnover is a rapid process as the half-life of HA molecules in the bloodstream is only 2–5 minutes.^7^ HA levels are significantly related to joint diseases. HA is the primary ligand for CD44 that is overexpressed in many cancer types including pancreatic, breast, lung, ovarian, and prostate.^8^ HA promotes tumor growth, metastasis and angiogenesis, which have shown the potential to be diagnostic and/or prognostic biomarkers in a few malignancies.^9^ HA family biomarkers promote bladder cancer (BCa) growth and progression very likely by upregulating the epithelial–mesenchymal transition (EMT) phenotype.^9^ Elevated HA in urine is an accurate diagnostic method for the efficient detection of BCa, regardless of tumor grade and stage.^10^ Recently, a research group determined HA by enzyme-linked immunosorbent assay^11^ and they showed the presence of this potential biomarker in the urine of high-risk patients for prostate cancer. Thus, HA showed a significant predictive ability for prostate cancer. With an optimal cut-off point of 50.13, HA has a 65% sensitivity and 53.9% specificity.^11^ Patients with HA levels more than 50.13 ng mL^−1^ had a 2.31 times greater likelihood of prostate cancer.^11^ A high level of HA can predict unfavorable breast cancer outcomes.^12^ Also, a high plasma level of HA is significantly associated with poor prognosis of metastatic breast cancer (MBC) patients. HA plasma levels decreased in parallel with the treatment response, signifying that HA could also be an effective biomarker for MBC treatment monitoring.^13^ HA accumulation has been found in different human digestive cancers and predicts advanced tumor stages, early recurrence, and poor prognosis of the patients. HA may be used to identify potentially high-risk groups of upper gastrointestinal cancers including gastric noncardia cancer and esophageal squamous cell carcinoma, particularly in high incidence areas.^14^ A high percentage of pericellular HA-positive cancer cells and a high intensity of the stromal HA signal correlate with lower cancer-related survival rates and recurrence-free rates among colorectal cancer (CRC) patients.^15^ However, there is a limited information about the prognostic value of serum HA levels in hepatocellular cancer (HCC) patients.^15^ Detection of special and sensitive biomarkers for every cancer is an urgent need, which could help postpone initiation of symptoms with early intervention, decreasing mortality rates, particularly for early diagnosis. HA could be a new prognostic biomarker useful for the selecting individual therapeutic strategies for cancer patients, therefore, early and sensitive detection of this marker is important for clinicians.

The increasing requirements for fast and reliable sensing devices stimulate the development of novel technological solutions, the conventional methods used for the bioassay of HA mainly include enzyme-linked immunosorbent assay (ELISA), a competitive fluorescence-based assay,^16^ plasmonic mass spectrometry,^17^ colorimetric, and immuno-electrophoresis.^18,19^ However, these methods have some limitations, such as requiring a time-consuming process, having laborious sample preparation steps, unsuitable for reusability, and being more expensive. Biosensors measure target molecules within a certain detection interval in a biological sample and are analytical measurement devices that transform this information into a meaningful electrical signal.^20^ Improving the performance of biosensors, particularly in clinical applications, is still urgently requested in this field. The development of biosensors has basically allowed simple, low-cost diagnostics and disease screening efficiently.^21^ Biosensors are being focused on developing highly sensitive and specific biomarker recognition to detect the disease at an early stage.^22^ Recently, a simple but sensitive electrochemiluminescence (ECL) biosensor has been designed for HAase detection based on the electrostatic interaction of anionic hyaluronic acid (HA) and cationic luminous reagent Ru(bpy)_3_^2+^. The proposed ECL system has been applied to detect HAase in urine samples with satisfying results and HAase inhibitors with high efficiency.^23^ A room-temperature phosphorescent (RTP) PDAD–Mn–ZnS QD biosensor was fabricated using poly (diallyl dimethylammonium chloride) (PDAD) as the modifier of MPA-capped Mn–ZnS QDs, and was used for HA determination. This proposed RTP sensor can avoid interference from matrix background fluorescence or stray light that occurs during fluorescence measurements. Therefore, this biosensor is potentially suitable for detecting HA in actual samples without complicated pretreatment.^24–28^

One of the most popular conductive oxides is ITO-based electrodes. Features that add to the popularity of this type of electrode are their transparency and excellent electrical properties.^28^ Additionally, a suitable surface, a wide working range, material stability, and linkage strength are their unique advantages.^29^ Also, the ITO-PET electrode is very cost-effective compared to other conventional working electrodes (GCE, Au, Pt, and dynamic.) In this study, we used a novel immunosensor based on ITO PET disposable electrode for the identification of HA using an electrochemical method.

We have developed a novel label-free electrochemical immunosensor based on modified ITO-PET (indium tin oxide-polyethylene terephthalate) electrodes for early detection of HA in real samples. ITO-based biosensors are utilized in many fields such as clinical diagnosis, and environmental monitoring.^25^ Using an ITO film, electrodes for biosensors have been capably increased because of their features, such as high electrical conductivity, low electrochemical background response, low cost, simple preparation for sensing applications, wide working area, corrosion resistance, and reusability.^26^ Until now, numerous biosensors have been developed by using ITO as a substrate for the working electrode, such as electrochemical nucleic acid biosensors, immunosensor, and microfluidic on-chip sensors.^25^ In this study, gold nanoparticles were chosen as the conductive material to modify an ITO electrode in order to enhance the electrical characteristics of the ITO substrate. Cyclic voltammetry (CV) and electrochemical impedance spectroscopy (EIS) techniques were used to investigate the electrochemical properties of the modified electrodes at each step of the biosensor fabrication and survey the formation of Ab–Ag immunocomplex, which is necessary for the recognition of HA in real samples. The field emission scanning electron microscopy (FESEM) technique was used for the characterization of the surface morphology of the biosensors as well. Reproducibility and repeatability and regeneration parameters were also examined for the determination of analytical characterization of the fabricated biosensor. To expound the binding characterization of HA and anti-HA, a single frequency impedance method was used as well.

## 2. Experimental

### 2.1. Materials

Five indium tin oxide coated polyethylene terephthalate film (ITO-PET) electrodes (thickness 0.175 mm, surface resistivity of 50 Ω cm^−2^) were purchased from NanoGostar Sepahan (Isfahan, Iran). Hyaluronic acid kit containing Biotin-HA-Ab and 320 ng mL^−1^ main concentration of HA-antigen was acquired from ZellBio GmbH (Germany) by Padgin Teb Company. Hydrogen tetrachloroaurate (iii) hydrate (HAuCl_4_–3H_2_O), cetyltrimethylammonium bromide (CTAB), and sulfuric acid, 98%, were obtained from ''Sigma-Aldrich (Ontario, Canada)'', for performing electrochemical deposition of Au nanoparticles on ITO-PET electrodes. An electrochemical investigation of the detection zone of the ITO-PETs was implemented using a ferricyanide/ferrocyanide solution including 0.5 M of K_4_Fe CN_6_/K_3_Fe CN_6_ along with KCl. Fresh frozen plasma samples were obtained from the Iranian Blood Transfusion Research Center (Tabriz, Iran). KCC-1-NH-CS_2_ was used for encapsulation and activation of biotinylated antibody that was synthesized by our research group, previously.^27^

### 2.2. Instrumentation

For electrochemical measurements, a three-electrode cell (from Metrohm), involving a Pt wire as a counter electrode, Ag/AgCl-saturated KCl as a reference electrode and ITO-PET as the working electrode was used, which was powered by an electrochemical system connecting the PalmSens system with PS4.F1.05 (Palm instruments, Utrecht, The Netherlands). The system was run on a PC using PSTrance 5.3 software. In this investigation, differential Pulse voltammetry with the current range of 100 pA–100 μA (*T*_equilibration_: 2 s*, E*_begin_: −1.0 V, *E*_end_: 1.0 V, *E*_step_: 0.1 V, *E*_pulse_: 0.005 V, *T*_pulse_: 0.2 s, scan rate: 0.1 V s^−1^), SWV with the current ranges of 10 nA–100 μA (*T*_equilibration_: 0 s, *E*_begin_: −1.0 V, −*E*_end_: 1.0 V, *E*_step_: 0.005 V, amplitude: 0.02 V, frequency: 10 Hz) and CV with the current ranges of 1 nA–100 μA (*T*_equilibration_: 0 s, *E*_begin_: −1.0 V, *E*_vertex1_: 1.0 V, *E*_vertex2_: −1.0 V, *E*_step_: 0.01 V, scan rate: 0.1 V s^−1^) techniques were applied for the primary detection of HA. The resistance investigation of each modified electrode was performed using the EIS technique with the current ranges of 100 pA–1 mA (*E*_dc_: 0.25 V, *E*_ac_: 0.01 V, max. frequency: 100 000 Hz, min. frequency: 0.1 Hz, *t* Max OCP: 50 s, stability criterion: 0.00001 mV s^−1^). The chronoamperometry technique (*T*_equilibration_: 2 s, *E*_dc_: −0.23 V, *t*_interval_: 0.1 s, *t*_run_: 120 s) was used for the electrochemical deposition of Au nanoparticles on ITO-PET electrodes. The surface morphology of the detection zone was recognized using high-resolution field-emission scanning electron microscopy (FE-SEM, Hitachi-Su8020, Czech) with a working voltage of 3–6 kV, and the chemical compounds of the electrodes were examined using energy-dispersive spectroscopy (EDS) along with FE-SEM.

### 2.3. ITO-PETs pre-treatment before electro-deposition

One of the most popular conductive oxides is the ITO-based electrode. Features that add to the popularity of this type of electrode are their transparency and excellent electrical properties.^28^ Additionally, a suitable surface, a wide working range, material stability, and linkage strength are their unique advantages.^29^ Also, the ITO-PET electrode is very cost-effective compared to other conventional working electrodes (GCE, Au, Pt, and dynamic.) In this study, we used a novel immunosensor based on the ITO-PET disposable electrode for the identification of HA using an electrochemical method. The first and most essential condition in the electrochemical experiment that affects the recognition process, is the modality and transparency of the active surface of ITO-PET electrodes. Contaminated environments and surfaces in such experiments affect the electrochemical reactions and as a result, cause incorrect and unwanted outcomes. Thus, a cleaned and prim surface is important before modification and bioanalysis. For this purpose, prior to the modification, the ITO surface was cleaned by sonication in acetone and distilled water for 10 minutes, then dried using nitrogen gas for 30 seconds.

### 2.4. Electrosynthesis of Au nanoparticles supported CTAB

Chronoamperometry is a suitable technique for depositing gold nanoparticles on ITOs. For this purpose, after the cleaning approaches, ITO-PETs were sliced into smaller pieces. Then, one side of the piece was insulated using a specific adhesive tape in order to save on nanoparticle consumption during deposition. The one-side insulated ITO-PET slice was translocated into the cell containing the Au nanoparticle solution, as the working electrode. After that, the electrodeposition was started following the CHA technique with the settings of *T*_equilibration_ = 2 s, *E*_dc_ = −0.23 V, *t*_interval_ = 0.1 s, *t*_run_ = 120 s. The deposition was performed on the uninsulated surface (Fig. S1 (see ESI†)).

Au nanoparticles were synthesized using CTAB and sulfuric acid. First, 10 mM ''HAuCl_4_–3H_2_O'' solution was prepared, then, CTAB 10% was dissolved in sulfuric acid at a concentration of 200 mM. After all, 10 mM of the “HAuCl_4_–3H_2_O” solution was added (With a ratio of 1 : 1 : 1) to the previously prepared solution. The container was shaken gently to obtain a homogeneous solution. The color of the solution was changed to orange. 10% CTAB solution was used as a pattern and capping agent to form a regular shape and protect the nanoparticles from agglomeration, also a sulfuric acid solution was used as a solvent solution.

### 2.5. KCC-1-NH-CS_2_ synthesis

**Fig. 1:**
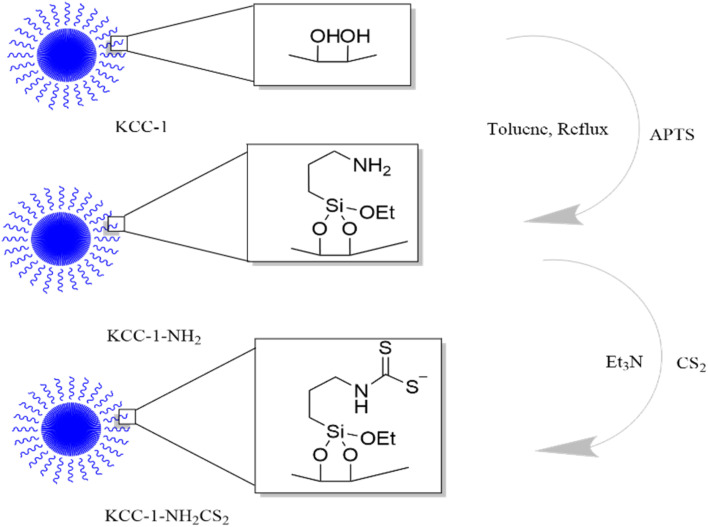
The synthesis procedure of KCC-1-NH-CS_2_.

KCC-1 and KCC-1-NH_2_ were synthesized following the technique described in our prior research.^30^ Dithiocarbamate-functionalized KCC-1-NH_2_ was prepared as follows: 100 mg of CS_2_ was dissolved in 20 mL of acetonitrile containing 1 percent Et_3_N and 90 mg of KCC-1-NH_2_. The resulting mixture was swirled overnight, to ensure complete dissolution. Finally, an evaporator was used to dry the solution (Fig. 1).

**Fig. 2:**
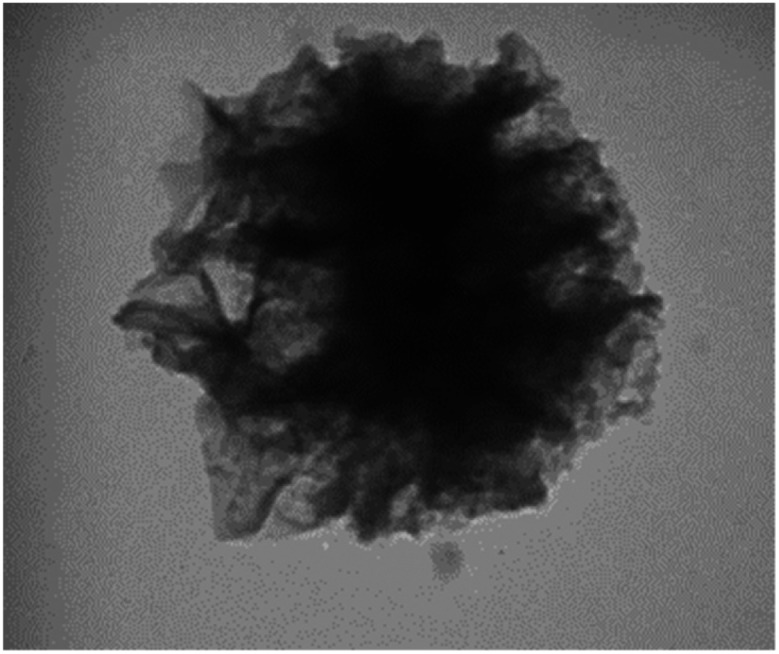
TEM images of KCC-1-NH-CS_2_ in various magnifications.

**Fig. 3:**
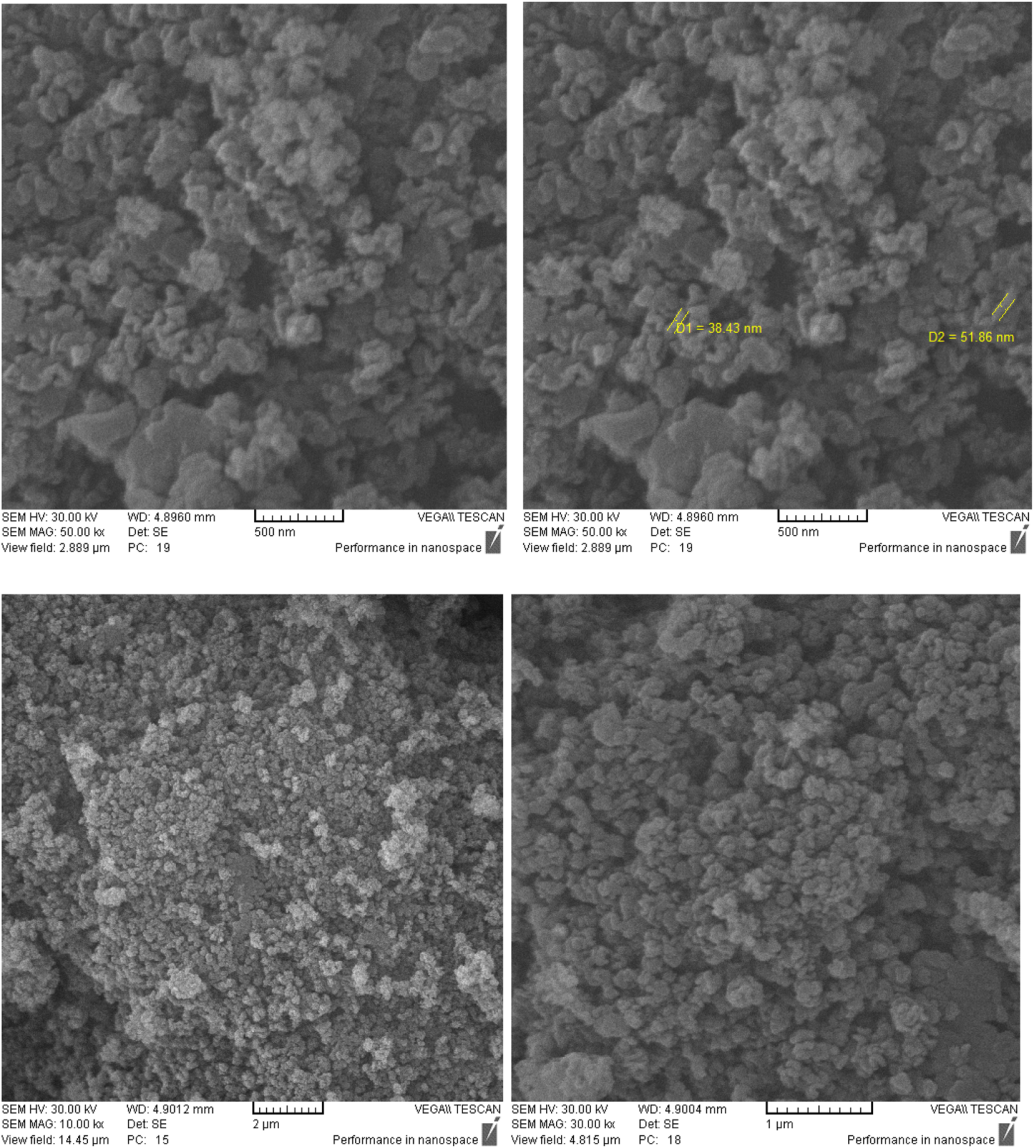
FESEM images of KCC-1-NH-CS_2_.

TEM was performed to identify the structure of KCC-1-NH-CS_2_. According to Fig. 2, the morphology of KCC-1-NH-CS_2_ is spherical and dendritic. Interestingly, some aggregation of these particles as particle-dendrites intertwine with other particle-dendrites was observed. For further investigation, FESEM was also performed on KCC-1, KCC-1-NH_2_, and KCC-1-NH-CS_2_. From the obvious deformation of particles after adding NH_2_ and CS_2_, the successful synthesis of KCC-1-NH-CS_2_ was confirmed. Moreover, the distinguishable smallest particles were determined to be 38.4 nm (Fig. 3).

**Fig. 4:**
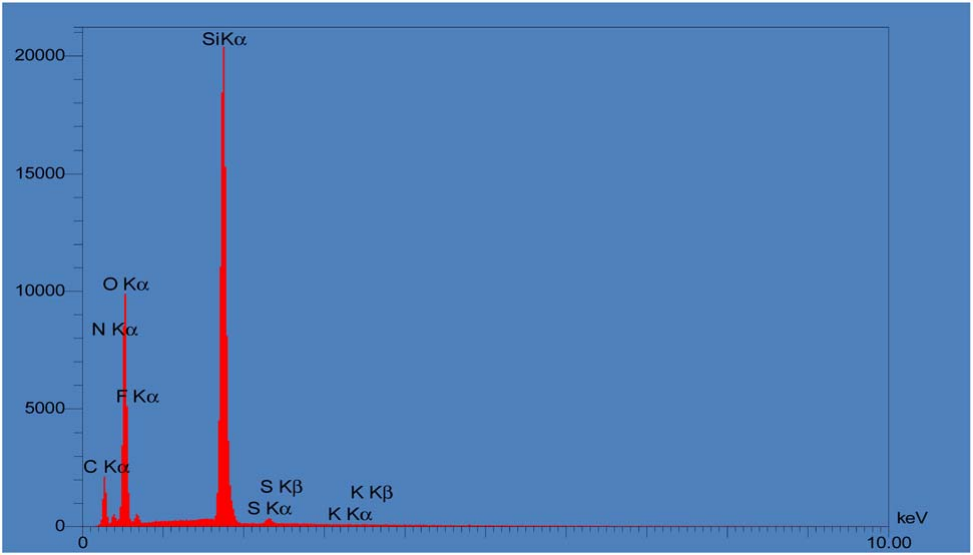
EDAX analyses of KCC-1, KCC-1-NH_2_ and KCC-1-NH-CS_2_.

Finally, EDAX was carried out for tracing the expected elements in KCC-1, KCC-1-NH_2,_ and KCC-1-NH-CS_2_. As shown in Fig. S3,† KCC-1 consisted mainly of O and Si. After functionalizing KCC-1 with NH_2_, and after that with CS_2_, amounts of N elements and S elements increased, respectively, which proved the formation of KCC-1-NH-CS_2_ (Fig. 4).

### 2.6. Co-encapsulation of Ab into KCC-1-NH-CS_2_

**Fig. 5:**
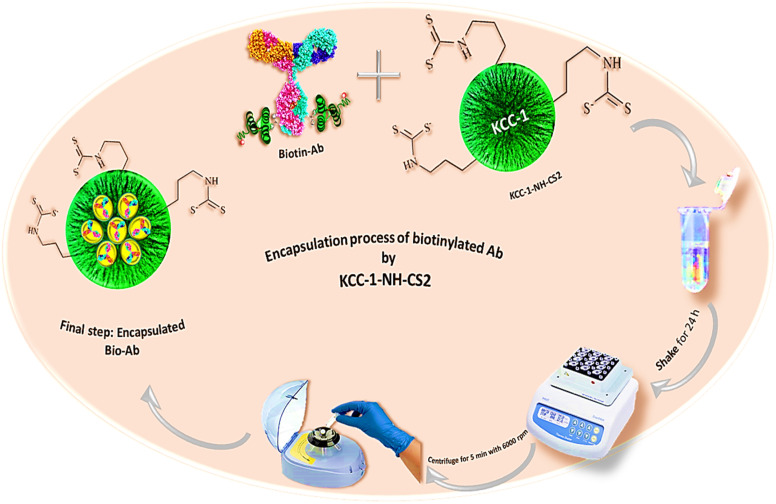
Schematic presentation for the encapsulation of biotinylated HA antibody on KCC-1-NH-CS_2_.

In this study, the possibility of encapsulating monoclonal antibodies within KCC-1-NH-CS_2_ nanoparticles was investigated. In this approach, polymeric nanoparticles (KCC-1-NH-CS_2_) were employed to encapsulate the HA antibody. As a result, biotinylated Ab, which was immobilized on the surface of the ITO-based electrodes, was trapped in KCC-1-NH-CS_2_. For this purpose, 0.001 g KCC-1-NH-CS_2_ was first dissolved in the phosphate buffer (pH 7.4). Then, 300 μL of biotinylated antibody was added to the above-mentioned solution and gently stirred for 24 hours at room temperature on a shaker. Therefore, after centrifuging the mixture at 6000 rpm for 5 minutes (Fig. 5), the supernatant was collected and kept at 4 °C until further use.

## 3. Fabrication of immunosensor

**Fig. 6:**
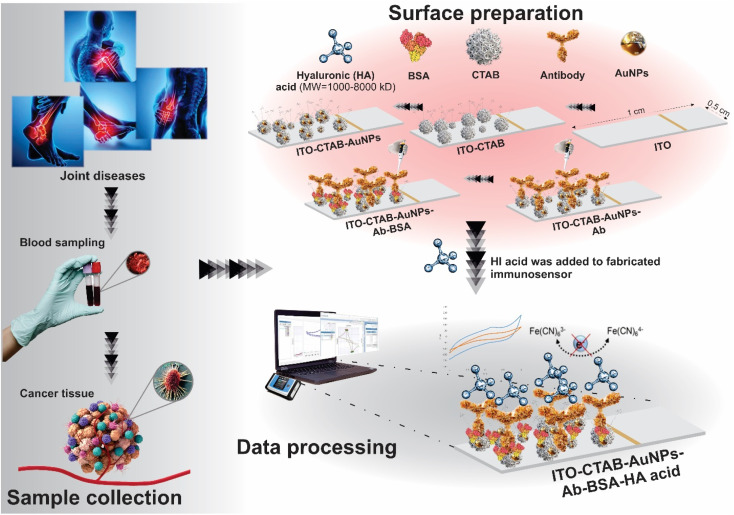
The main steps in creating HA immunosensor.

After depositing Au on ITO-PET electrodes, it took 20 minutes for the surface to dry completely. Then, 5 μL of HA Biotin-Ab that was encapsulated and activated with KCC-1-NH-CS_2_ solution was immobilized in the detection zone (ITO-PET-Au) and incubated for 3 hours at a temperature of 22 °C because the nanocomposite is sensitive to temperatures above 22 °C. Phosphate buffer (with pH 7.4) was used for preparing the KCC-1-NH-CS_2_ solution. In order to encapsulate and activate biotinylated Ab, 100 μL of phosphate buffer was combined with 0.001 g of KCC-1-NH-CS_2_ and added to 300 μL of antibody, placed on a shaker for 24 hours at room temperature. Then, centrifuged for 5 minutes at 6000 rpm and the resulting supernatant was used for investigations. After incubation of Ab, 5 μL of BSA protein solution (10%) was drop-casted on the modified detection zone (ITO-PET-Au-Ab) as a blocking agent and incubated for 40 minutes. Finally, 5 μL of HA antigen was immobilized on ITO-PET-Au-Ab-BSA, this step lasted for 2 hours. After washing the modified surface with DW, it was translocated into an electrochemical cell containing K_4_Fe(CN)_6_/K_3_Fe (CN)_6_/KCl 0.5 M for electroanalysis. Fig. 6 illustrates all the preparation steps of the immunosensor.

### 3.1. Electrochemical behavior of different steps of fabricated immunosensor

**Fig. 7:**
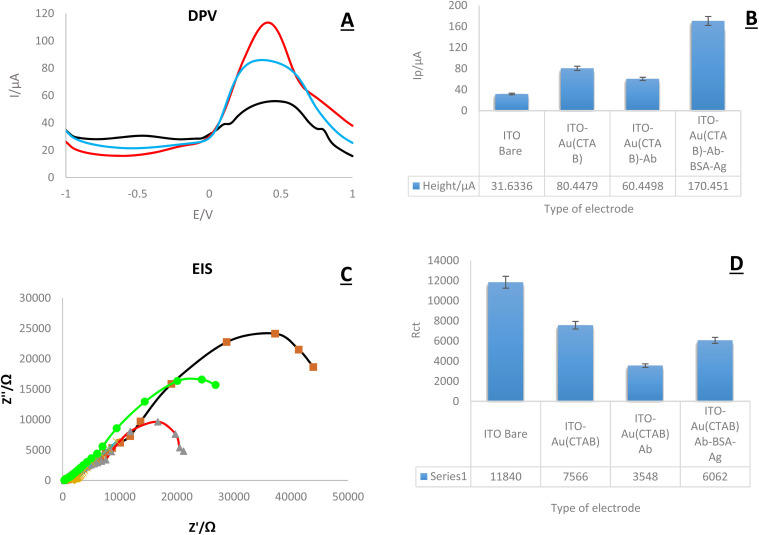
(A and D) DPV and EIS of immunosensor fabrication steps (Bare ITO-PET electrode, ITO-PET-AuNPs (CTAB), ITO-PET-AuNPs (CTAB)-Ab (encapsulated by KCC-1-NH-CS_2_), ITO-PET-AuNPs (CTAB)-Ab-BSA-HA. Data information of DPV: (*T*_equilibration_: 2 s, *E*_begin_: −1.0 V, *E*_end_: 1.0 V, *E*_step_: 0.1 V, *E*_pulse_: 0.005 V, *T*_pulse_: 0.2 s, scan rate: 0.1 V s^−1^), and EIS: (*E*_dc_: 0.25 V, *E*ac: 0.01 V, max. frequency: 100 000 Hz, min. frequency: 0.1 Hz,*t*_Max_ OCP: 50 s, stability criterion: 0.00001 mV s^−1^), in the presence of K_4_Fe(CN)_6_/K_3_Fe (CN)_6_/KCl 0.5 M. (B and D) DPV and EIS histograms of *versus* modifications of ITO-PET electrode, respectively. (RSD = 0.698%, 0.479%, respectively, *n* = 4).

In this part, research electrochemical behavior of the created immune platform was evaluated. For this purpose, DPV and EIS techniques were applied in the presence of K_4_Fe(CN)_6_/K_3_Fe (CN)_6_/KCl 0.5 M (Fig. 7). As the results show, the lowest peak current is related to the bare electrode. The highest current intensity was recorded in the presence of AuNPs and the antigen. According to the current intensity, the highest peak current in both the DPV and EIS diagrams is related to the electrode modified with Ag-Ab-AuNPs, and the lowest peak height is related to the bare electrode. The obtained results were expected and are consistent with previous studies. Numerous studies have shown that gold nanoparticles significantly increase electrical conductivity and subsequently increase current intensity.^31^

### 3.2. Morphological study of used nanocomposite

**Fig. 8:**
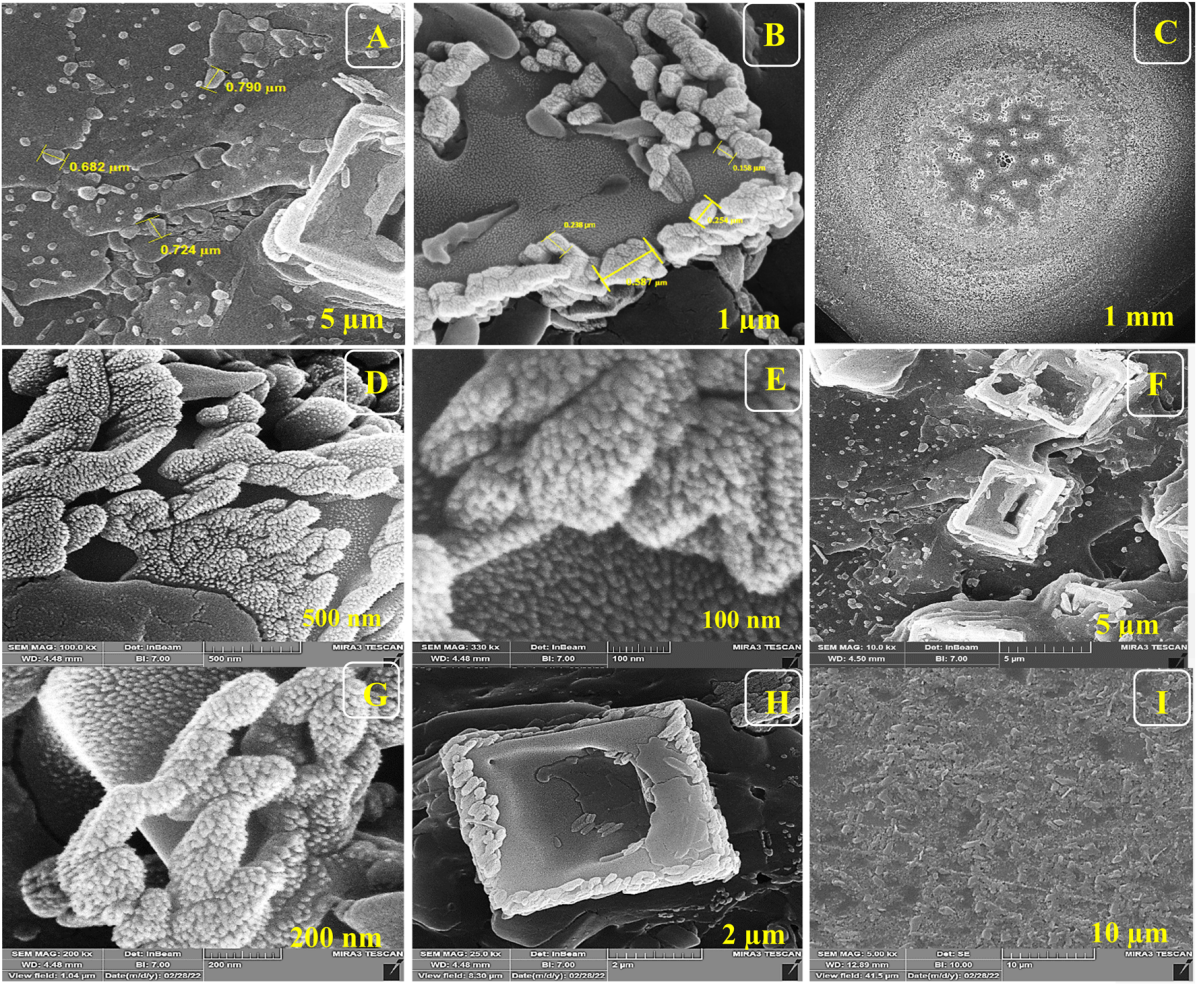
(A–I) FE-SEM of ITO-PET modified AuNPs(CTAB) under different magnifications. ITO-PET-AuNPs(CTAB) were evaluated in the second step.

**Fig. 9:**
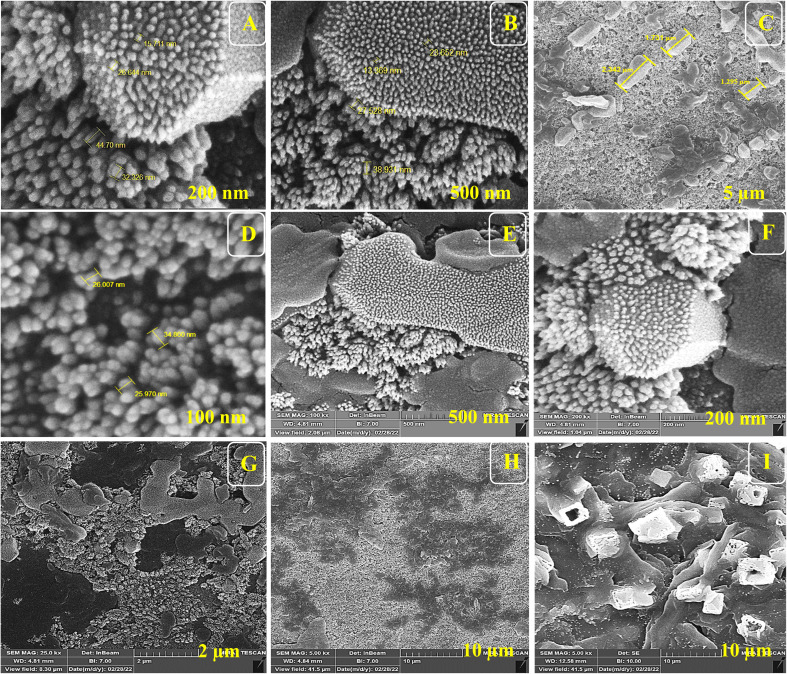
(A–I) FE-SEM of ITO-Au(CTAB)-Ab (Ab encapsulated by KCC-1-NH-CS_2_) under different magnifications.

**Fig. 10:**
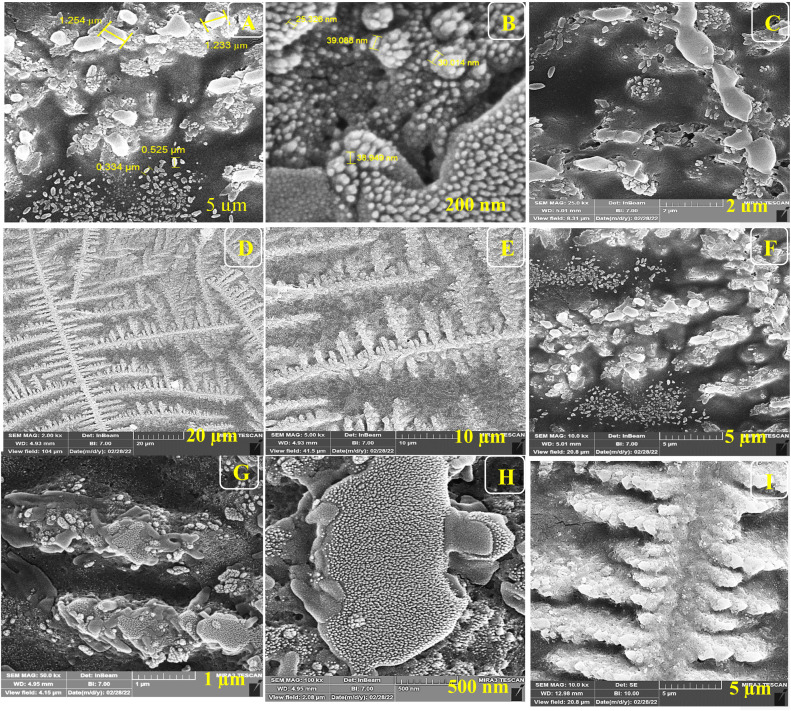
(A–I) FE-SEM of ITO-PET-AuNPs(CTAB)-Ab-BSA (Ab encapsulated by KCC-1-NH-CS_2_) under different magnifications.

**Fig. 11:**
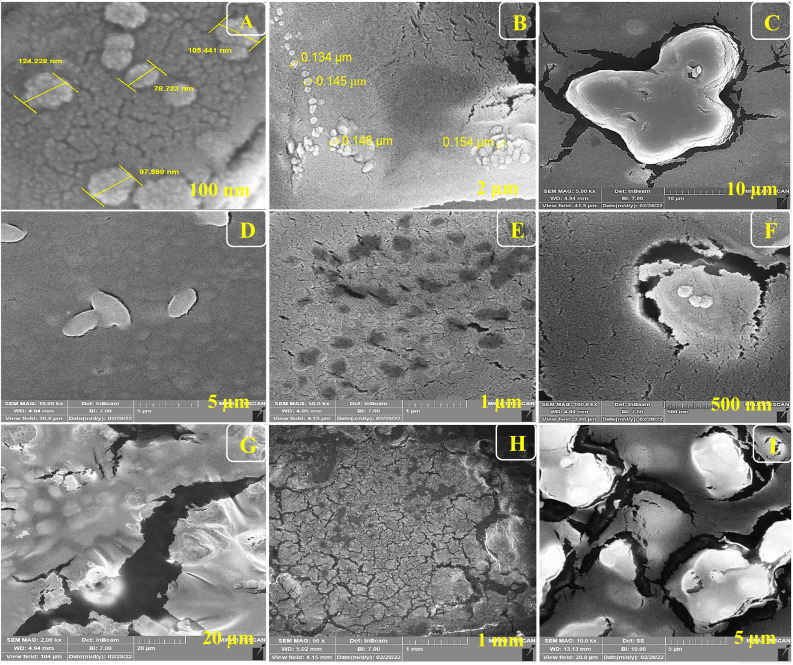
(A–I) FE-SEM of ITO-PET-AuNPs(CTAB)-Ab-BSA-Ag (Ab encapsulated by KCC-1-NH-CS_2_) under different magnifications.

For the morphological study of the synthesized nanocomposite under different stages, FE-SEM was used appropriately. Firstly, ITO-PET-AuNPs(CTAB) were selected for the analysis of the morphology of the nanocomposite. As shown in Fig. 8, the nanocomposites have a special arrangement at this stage and are placed next to each other. Also, Fig. 9 indicates that the antibody was successfully entrapped in KCC-1-NH-CS_2_ and efficiently immobilized on the surface of ITO-PET modified AuNPs(CTAB). The addition of Ab seems to play an important role in its formation. Evaluation of ITO-PET-Au(CTAB)-Ab-BSA (Fig. 10) was the third step in imaging. The arrangement of the nanocomposite at this stage is almost the same as the previous stage, so the nanoparticles are compactly placed next to each other. The last imaging step was the evaluation of ITO-PET-AuNPs(CTAB)-Ab-BSA-Ag (Fig. 11). At this stage, the material created flower-like cosmetic particles. The addition of antigens can be the main reason for this type of makeup. In general, the results obtained from FE-SEM show that nanoparticles can have different shapes and arrangements at different stages. The different structures of nanocomposites at different stages are the main reason for this morphological difference, which was fully concluded in this study.

As shown in Fig.6, the nanocomposites have a special arrangement at this stage and are placed next to each other. Also, Fig.6 indicates that the antibody was successfully entrapped in KCC-1-NH-CS_2_ and efficiently immobilized on the surface of ITO-PET modified AuNPs(CTAB). The addition of Ab seems to play an important role in its formation. Evaluation of ITO-PET-Au(CTAB) -Ab-BSA was the third step in imaging.

The arrangement of the nanocomposite at this stage is almost the same as the previous stage so the nanoparticles are compactly placed next to each other. The last imaging step was the evaluation of ITO-PET-AuNPs(CTAB) -Ab-BSA-Ag.

### 3.3. Energy-dispersive spectroscopy and MAP analysis

**Fig. 12:**
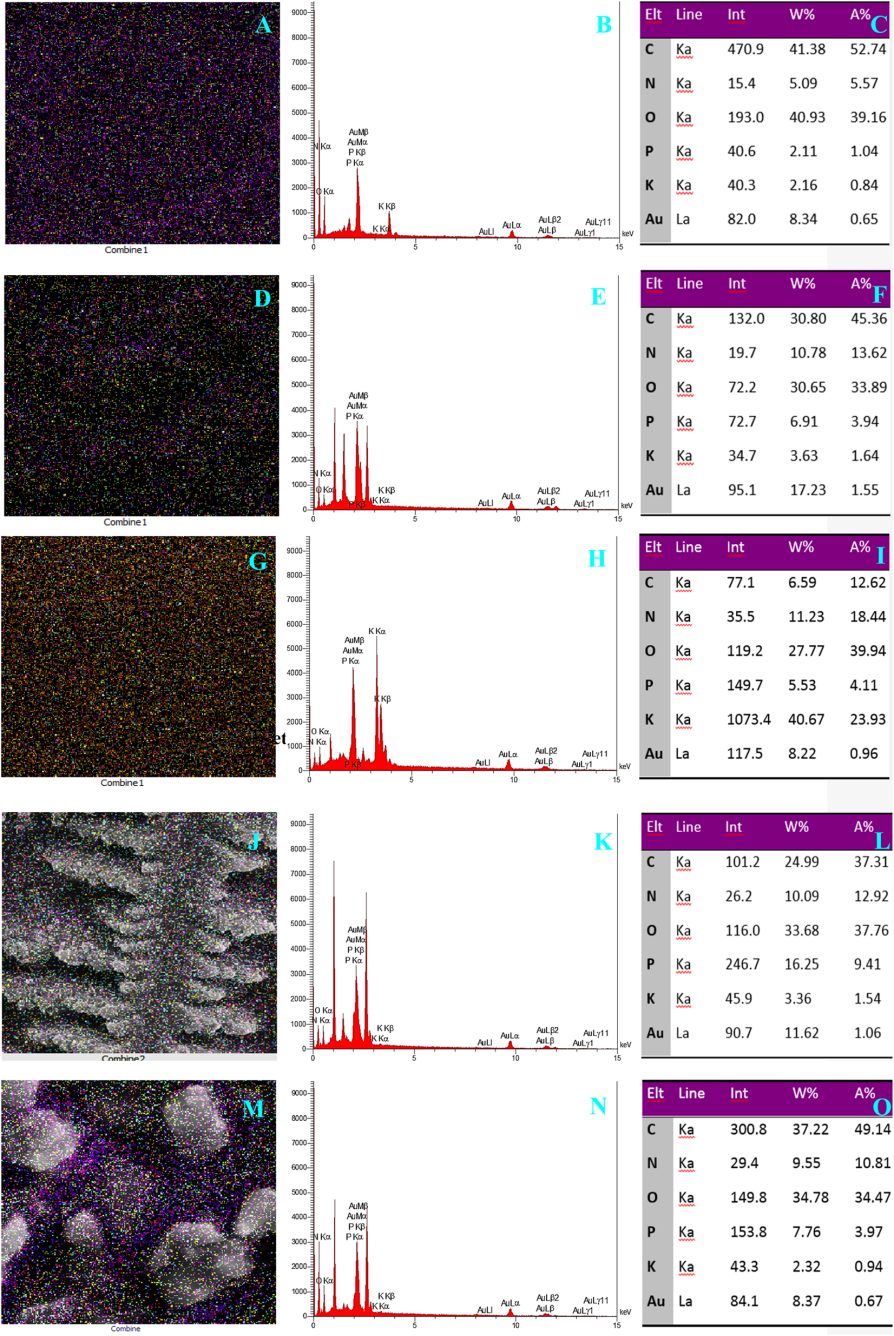
(A–O) Atomic-resolution chemical mapping using energy-dispersive X-ray spectroscopy of different steps of the fabricated immunosensor: (A–C) ITO-PET bare. (D–F) ITO-PET modified AuNPs(CTAB). (G–I) ITO-PET modified AuNPs(CTAB)-Ab. (J–L) ITO-PET modified AuNPs(CTAB)-Ab-BSA. (M–O) ITO-PET modified AuNPs(CTAB)-Ab-BSA-HA.

EDS and MAP analysis were applied in this work towards elementary discovery. Atomic-resolution chemical mapping using energy-dispersive X-ray spectroscopy of different steps of the fabricated immunosensor revealed valuable data. The analysis of carbon, oxygen, potassium, phosphor, nitrogen, and gold elements was investigated. A–C figures are related to ITO-PET bare electrode (Fig. 12). As can be seen, C and O are in the highest amounts in this step. The maps acquired at this stage are completely meaningful and based on the percentage of different elements. Each color point corresponds to a specific element. According to the obtained results, the purple dots are related to carbon, and as shown in the pictures, purple dots are dominant in the presence of a high percentage of carbon. The change in the percentage of elements in different stages changes the number of colored dots in the map, so the presence of AuNPs has increased the yellow and blue dots (D–O). After the addition of antigen and antibody in the stage of creating the biosensor, in addition to changing the color points, the morphological arrangement of the elements also shows a significant change (J–O). In general, the results obtained at this stage show that the arrangement of nanoparticles in the structure of biosensors depends on the presence of different elements and any change in their weight percentage will cause morphological changes.

## 4. Results and discussion

### 4.1. Analytical study

**Fig. 13:**
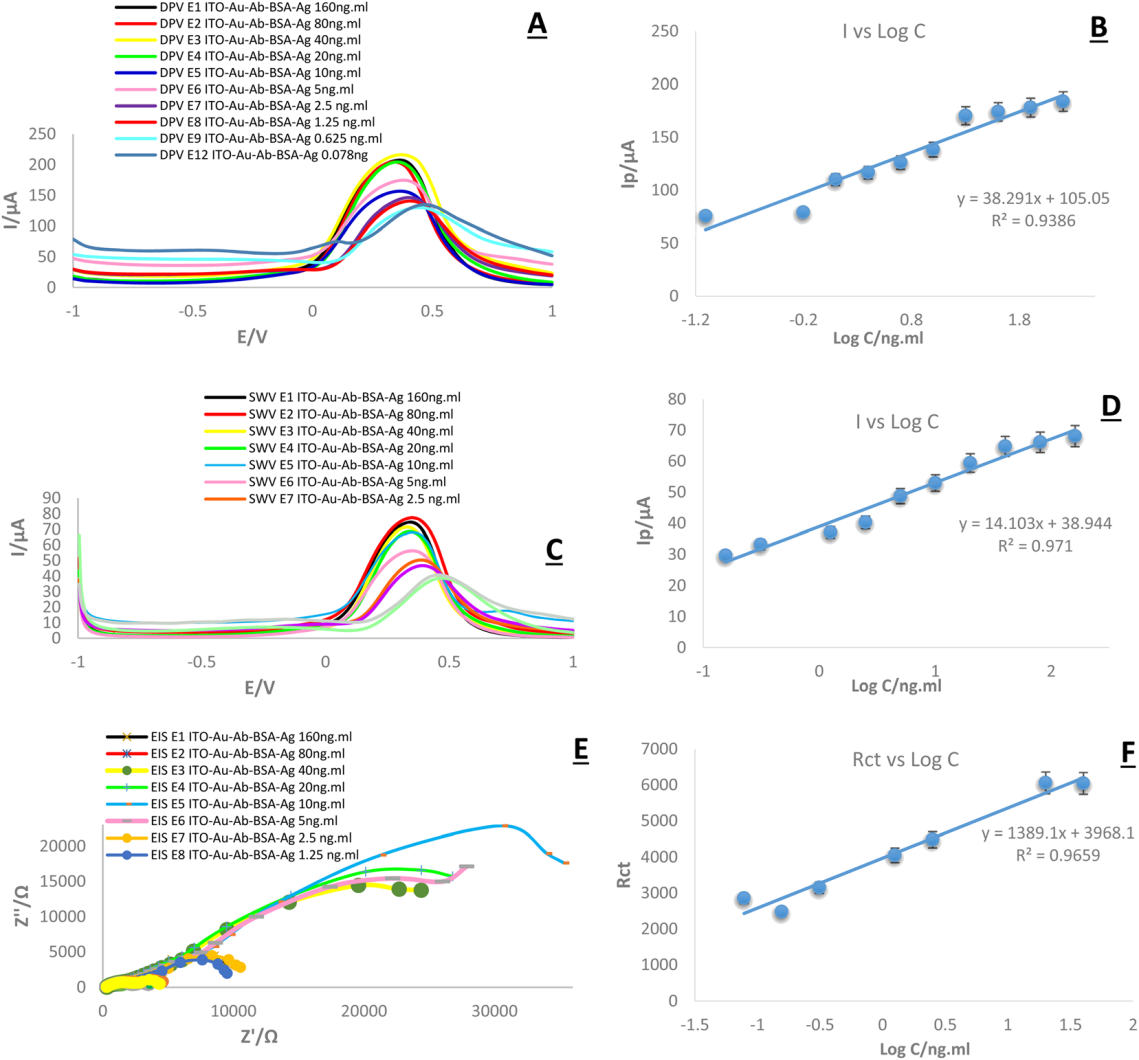
(A, C and E) DPV, SWV and EIS of the fabricated immunosensor in the presence of HA at different concentrations (160, 80, 40, 20, 10, 5, 2.5, 1.25, 0.625, 0.312, 0.156, 0.078 ng mL^−1^), in the presence of K_4_Fe(CN)_6_/K_3_Fe(CN)_6_ 0.5 M containing KCl (0.1 M). (B, D and F) Calibration curves of ITO-PET-AuNPs(CTAB)-Ab-BSA modified HA immunosensor in different concentrations. (RSD = 0.299%, 0.287%, 0.35%, *n* = 10, *n* = 10, *n* 0 = 7, for DPV, SWV and EIS, respectively).

In this research, DPV, SWV, and EIS were applied for the bioanalytical study of the immunosensor toward the measurement of HA. This trend is important and predictable, as the current intensity decreases with a decrease in the concentration and subsequent changes in peak height, as seen in the DPV and SWV plots (Fig. 13). In other words, the highest altitude refers to the highest concentration and the lowest altitude refers to the lowest concentration. However, there was no noticeable change in the locations of the peaks.

### 4.2. Analytical study of a real sample (plasma)

**Fig. 14:**
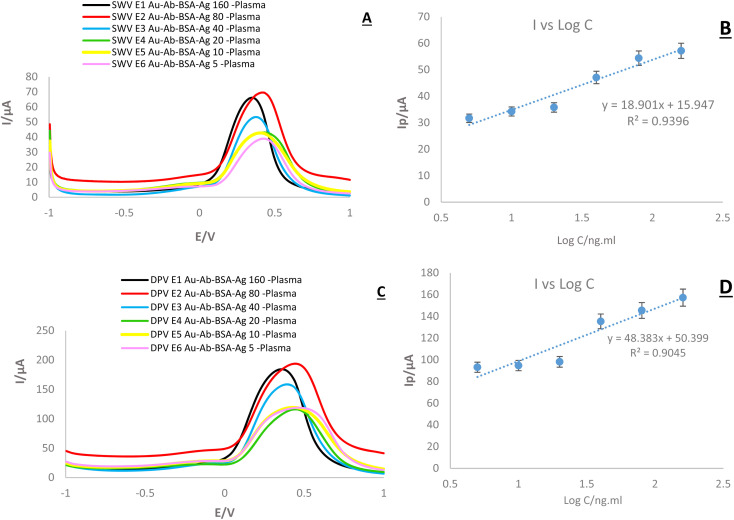
(A and C) SWV and DPV of the fabricated immunosensor in the presence of real sample (human plasma) in the concentrations (160, 80, 40, 20, 10, 5), in the presence of K_4_Fe (CN)_6_/K_3_Fe (CN)_6_ 0.5 M containing KCl (0.1 M). (B and D) calibration curves (RSD = 0.25%, 0.24%, *n* = 6).

One of the most important and central parts of this study was to assess the sensitivity of created immuno-platform in connection with real samples. Similar to the previous step, DPV and SWV techniques were applied for the discovery of real sample analyses. The results (Fig. 14) show that the designed system follows the expected diagnostic process. The most important achievement of this stage is matching the results of the SWV, DPV, and EIS techniques. In all three techniques used in this stage, simultaneously with the decrease in antigen concentration, the current intensity and then the peak height decreased. Considering the stability of the conditions with all three techniques, it can be claimed that the changes recorded in the graphs are only related to the antigen concentration. In this part, log concentration values show the valuable data. As revealed, the highest current (180 μA in DPV and 69 μA in SWV) was recorded for the highest concentration (log C (ng mL)^−1^). There is also a clear linear relationship between different concentrations. The linear relationship can be expressed by the recoded regression equation in the figures. The results (Fig. 14) demonstrate that the designed system follows the expected diagnostic process. The main achievement of this stage is matching the results of SWV, and DPV techniques. In both techniques used at this stage, simultaneously, with the decrease in antigen concentration, the current intensity and then the peak height decreased. Since the stability of the conditions with both techniques, it can be claimed that the changes recorded in the graphs are only related to the antigen concentration. In this part, log concentration values demonstrate valuable data. As discovered, the highest current (200 μA in DPV and 70 μA in SWV) was recorded for the highest concentration (log C/(ng ml)^−1^). There is also a clear linear relationship between different concentrations. The linear relationship can be expressed by the recoded regression equation in the figures. Comparing the graphs obtained in the previous step with the graphs of a real sample shows that the current intensity is slightly higher in relation to a real sample, and this difference can be related to the nature of the sample (Table 1).

**Table 1:** The developed methods for determination of the HA (MW = 1000–8000 kDa)

Detection method	Sample	Detection time	Stability	Detection range	Limit of detection	Advantages/limitation	Ref.
Phosphorescence biosensor based on QDs	Sodium hyaluronate eye drops	15 min	60 min	0.08–2.8 μg mL^−1^	0.03 μg mL^−1^	Rapid and sensitive	24
Fluorescence assay	Human serum	Lower than 5 h	<5 h	0.2 and 500.0 μg L^−1^	0.2 μg L^−1^	Superior image clarity over fluorescence microscopy/prolonged exposure to fluorescent light can result in bleaching and loss of fluorescence intensity	32
CTAB turbidimetric method	Fermentation broth samples	15 min	—	20–160 μg mL^−1^	1.55 μg mL^−1^	Turbidity measurement can be performed without destroying the sample./need for more samples	33
HPLC-UV	Pharmaceutical formulations	60 min	—	320–480 μg mL^−1^	320 μg mL^−1^	Complexity, expensive, time consuming	34
Turbidity assay	Cultured broth	30 min	—	2.35 ± 0.04 mg mL	10 mg mL	Extremely laborious as well as time-consuming	35
ELISA	White rabbits' blood	70 min	70 min	28 ± 17 ng mL	28 ng mL	High specificity/sophisticated techniques and expensive culture media are required	36
Rayleigh resonance scattering (RRS)	Eye drop	—	≤2h	0.4–48.0 μg mL^−1^	0.096 μg mL^−1^	Rapid/low sensitivity	37
Colorimetric enzyme-coupled assay	Complex samples	25–60 min	70 min	3–2000 mg L	0.3 mg L	Simple operation, quick response, adaptable sensitivity/fairly expensive, some surfaces reflect light, making it difficult to take measurements, cannot be used as colorless compounds	19
Electrochemical immunosensor	Real samples	1 h	4 h	0.078–160 ng mL	0.078 ng mL	High sensitivity, low cost	This work

### 4.3. Stability of biosensor substrate

Stability is one of the key features of biosensors' performance that requires continuous monitoring. Stability is the degree of susceptibility of biosensors to internal and external environmental hazards. Factors that affect stability are bioreceptor affinity (degree of binding of the analyte to the bioreceptor) and bioreceptor degradation over time.^38^ The CV technique was used to evaluate the stability and as shown in Fig. S2 (see ESI†), the stability of the created biosensor follows a certain process. The results show that the current intensity on the first day is 200 μA, while these data decreased to 172 μA after 24 hours, and finally, these data decreased to 58 μA after 48 hours. Based on the obtained results, the prepared substrate (AuNPs (CTAB)) is stable for about 48 h, which is suitable for the next investigation. In this study, after evaluating the inter-day stability of the electrode substrate (ITO-PET-AuNPs (CTAB)), the intra-day stability was also measured Fig. S3 (see ESI†). The results (Fig. S3 (see ESI†)) show that the stability of the created system between 0–4 hours has a decreasing trend, although this trend is not significant. Similar to that in the previous section, the narrowest graph corresponds to the longest time of stability (4 hours). Based on the obtained results, suitable intra-day stability was obtained for the provided substrate.

### 4.4. Investigation of reproducibility and repeatability

Reproducibility is the ability of the biosensor to generate identical responses for a duplicated experimental set-up. Reproducibility is characterized by the precision and accuracy of the transducer in a biosensor.^39^ Ideal reproducibility is one of the most important features of the system designed in this study. As can be seen in the results (Fig. S4 (see ESI†)), the biosensor exhibits almost the same electrochemical behavior under the same conditions, both in terms of current intensity and location of peaks. As the graph shows, a peak height of 400 μA was recorded in all three cases, confirming the ideal reproducibility of the developed biosensor. Similar results were obtained by the CV technique, which confirmed SWVs data. Standard deviation is the degree of dispersion or the scatter of the data points relative to its mean, in descriptive statistics. This method revealed that the values are spread across the data sample and it is the measure of the variation of the data points from the mean. The standard deviation of a sample, statistical population, random variable, data set, or probability distribution is the square root of its variance. In this part of the research, the standard deviation was measured for three electrodes and as shown in Fig. S4 (see ESI†), SD = 1.37 (*n* = 3) for the SWV technique and SD = 1.21 (*n* = 3) for the CV technique were recorded. Also, to confirm the proper reproducibility of the system developed in this study, the repeatability of the biosensor was evaluated using the CV technique. The results (Fig. S5 (see ESI†) obtained in this step confirm the proper repeatability of the immunosensor and as recorded in the obtained graphs, the height and location of the peaks are almost the same, on the other hand, the width of the peaks does not show a significant change. The error bars represent the standard deviations of three electrodes of each sample within the same experiment (SD = 5.17, SD = 4.51, SD = 6.08). Based on the acquired results, the standard deviation recorded for three electrodes under the same conditions is almost equal and the difference obtained is not significant.

### 4.5. Selectivity

Selectivity is perhaps the most important feature of a biosensor. Selectivity is the ability of a bioreceptor to detect a specific analyte in a sample containing other admixtures and contaminants. The best example of selectivity is depicted by the interaction of an antigen with the antibody.^40^ The results (Fig. S6 (see ESI†) of the selectivity confirm the important point that the created biosensor has a suitable selectivity in detecting the target antigen among CEA, PSA, and CA-15. In other words, the designed system is able to differentiate HA specifically. According to the recorded graphs, the HA diagram differs from other antigens in terms of height, location, and peak width. With more details, the height and location of the peak in relation to HA are 210 μA and 4.4 V, respectively, while in relation to other interfering antibodies, the peak height for CEA, CA-15, and PSA are 115, 170, and 180 (μA), respectively. Also, the peak location has changed accordingly.

### 4.6. Kinetic study

Cyclic voltammetry was used to evaluate the electrode-catalytic behavior of AuNPs-CTAB electrodes and the redox behavior of K_4_Fe(CN)_6_/K_3_Fe(CN)_6_. The results of CV as a function of the scan rate of the AuNPs-CTAB electrodes are shown (Fig. S7 ((see ESI†))). The anode and cathode peaks correlate with the oxidation and reduction of the K_4_Fe(CN)_6_/K_3_Fe(CN)_6_ redox couple. The anode and cathode peak currents were found to increase linearly at different potential sweep rates in the range of 10 to 1000 mV s^−1^, confirming the diffusion-limiting behavior of the redox couple electrochemical reactions. It can be concluded that a slow sampling rate slows electron exchange on the electrode surface, records peak currents, shortens peak currents, and narrows the voltammogram. From the linear dependence of the peak current on the square root of the sampling rate and the Napier logarithm of the peak current on the Napier logarithm of the sampling rate. We conclude that the reaction is diffusion controlled.

## 5. Conclusion

In this work, a label-free electrochemical immunosensor for the sensitive and specific detection of HA was designed by using transparent ITO-PET electrodes. The resulting immunosensor has various advantages, including high sensitivity, good selectivity, long storage stability, excellent reproducibility, and simplicity to fabricate and function. Antibody encapsulation inside KCC-1-NH-CS_2_ was reported for the first time in this study. In this work, KCC-1-NH-CS_2_ acts as a green, efficient, and reusable nanomaterial. In addition, the developed HA-based immunosensor has suitable LLOQ “(0.078 ng mL)” and has a wide linear detection range “(0.078 to 160 ng mL^−1^)”. In summary, we developed an HA-based biosensor that is regenerative, sensitive, practical, low-cost, and has a wide detection range. It has considerable potential in cancer biomarker diagnosis.

## Abbrevations

HA: Hyaluronic acid
ITO-PET: Indium tin oxide-polyethylene terephthalate
AuNPs: Gold nanoparticles
EIS: Electrochemical impedance spectroscopy
CV: Cyclic voltammetry
FESEM: Field emission scanning electron microscopy
CTAB: Cetyltrimethylammonium bromide
CD: Cluster of differentiation
HAases: Hyaluronidases
ECM: Extracellular matrix
GAGs: Glycosaminoglycans
HMW: High molecular weight
LMWHA: Low molecular weight HA
BCa: Bladder cancer
EMT: Epithelial–mesenchymal transition
MBC: Metastatic breast cancer
CRC: Colorectal cancer
HCC: Hepatocellular cancer
ELISA: Enzyme-linked immunosorbent assay
ECL: Electrochemiluminescence
RTP: Room-temperature phosphorescent
PDAD: Poly (diallyldimethylammonium chloride
CA15-3: Cancer antigen 15-3

## Data availability

Data sharing is not applicable. It will be shared if needed.

## Author contributions

Conceptualization: Sanam Dolati and Mohammad Hasanzadeh data curation: Fereshteh Kohansal formal analysis: Fereshteh Kohansal funding acquisition: Sanam Dolati investigation: Ahmad Mobed methodology: Mohammad Hasanzadeh project administration: Sanam Dolati Software: Fereshteh Kohansal supervision: Sanam Dolati and Mohammad Hasanzadeh validation: Mohammad Hasanzadeh, Writing–original draft: Ahmad Mobed and Sanam Dolati, writing –review and editing: Mohammad Hasanzadeh.

## Conflicts of interest

The authors declare that they have no known competing financial interests or personal relationships that could have appeared to influence the work reported in this paper.

## Supplementary Material

RA-012-D2RA04984H-s001

RA-012-D2RA04984H-s002

RA-012-D2RA04984H-s003

RA-012-D2RA04984H-s004

RA-012-D2RA04984H-s005

RA-012-D2RA04984H-s006

RA-012-D2RA04984H-s007

RA-012-D2RA04984H-s008

RA-012-D2RA04984H-s009

RA-012-D2RA04984H-s010

RA-012-D2RA04984H-s011
