# Supplementary material for: A novel immuno-device based on the specific binding of AuNP-supported CTAB with biotinylated antibody of hyaluronic acid toward an early-stage recognition of a biomarker: a bioanalytical assay in real samples using disposal biosensor technology
Source: RSC Adv. 2022 Oct 5;12(44):28473–88. doi: 10.1039/d2ra04984h (PMC9533320; doi:10.1039/d2ra04984h)
Supplement: RA-012-D2RA04984H-s001 [file RA-012-D2RA04984H-s001.pdf]

Electronic Supplementary Material (ESI) for RSC Advances.
This journal is © The Royal Society of Chemistry 2022

Supporting information

A novel immuno-device based on specific binding of AuNPs supported CTAB with biotinylated antibody of Hyaluronic Acid toward early-stage recognition of biomarker: A bioanalytical assay in real samples using disposal biosensor technology

Ahmad Mobed [abc1], Fereshteh Kohansal [c1], Sanam Dolati [b*], Mohammad Hasanzadeh [c**]

[a] Aging Research Institute, Faculty of Medicine, Tabriz University of Medical Sciences, Iran

[b] Physical Medicine and Rehabilitation Research Center, Aging Research Institute, Tabriz University of Medical Sciences, Tabriz, Iran

[c] Pharmaceutical Analysis Research Center, Tabriz University of Medical Sciences, Tabriz, Iran

Corresponding authors
* Physical Medicine and Rehabilitation Research Center, Aging Research Institute, Tabriz University of Medical Sciences, Tabriz, Iran. sanam.dolati@gmail.com

** Pharmaceutical Analysis Research Center, Tabriz University of Medical Sciences, Tabriz 51664, Iran. hasanzadehm@tbzmed.ac.ir

[1]Co-first author

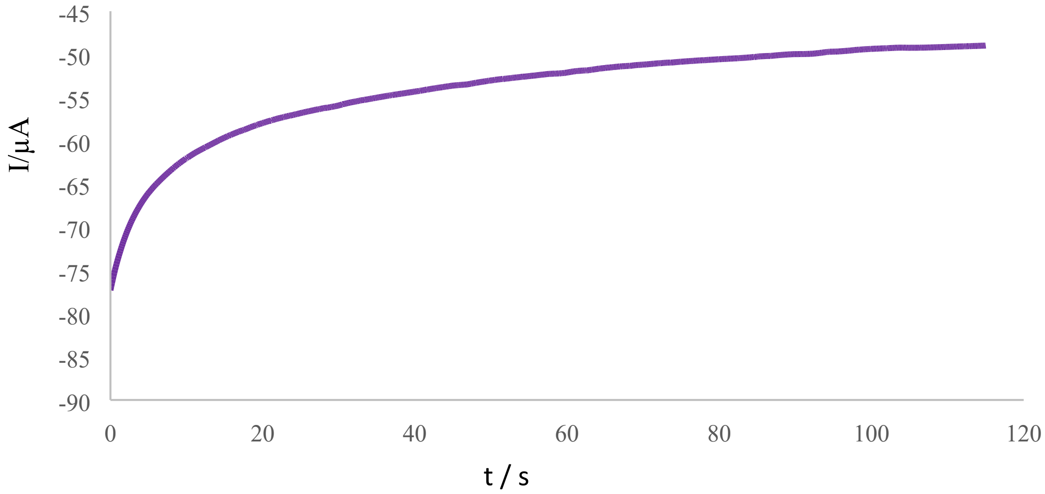

Fig. S1. Deposition of Au(CTAB) on ITO-PET by CHA technique, with the data's of: $T_{equilibration}$ = 2 s, Edc= -0.23 V, $t_{interval}$ = 0.1 s, t run=120 s.

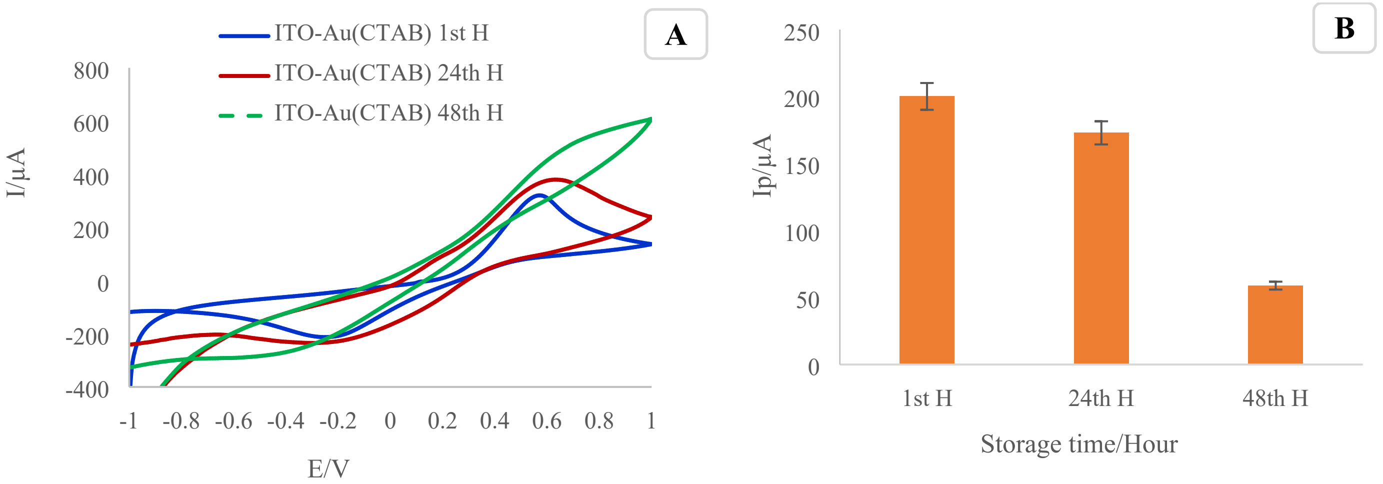

Fig. S2. A) CVs of ITO-PET-AuNPs (CTAB) in different times of storage (1st, 24th and 48th hour), in the presence of $K_4Fe(CN)_6$ /$K_3Fe(CN)_6$ 0.5M containing KCl. **B)** Histogram of peak current in different storage times (per hour).

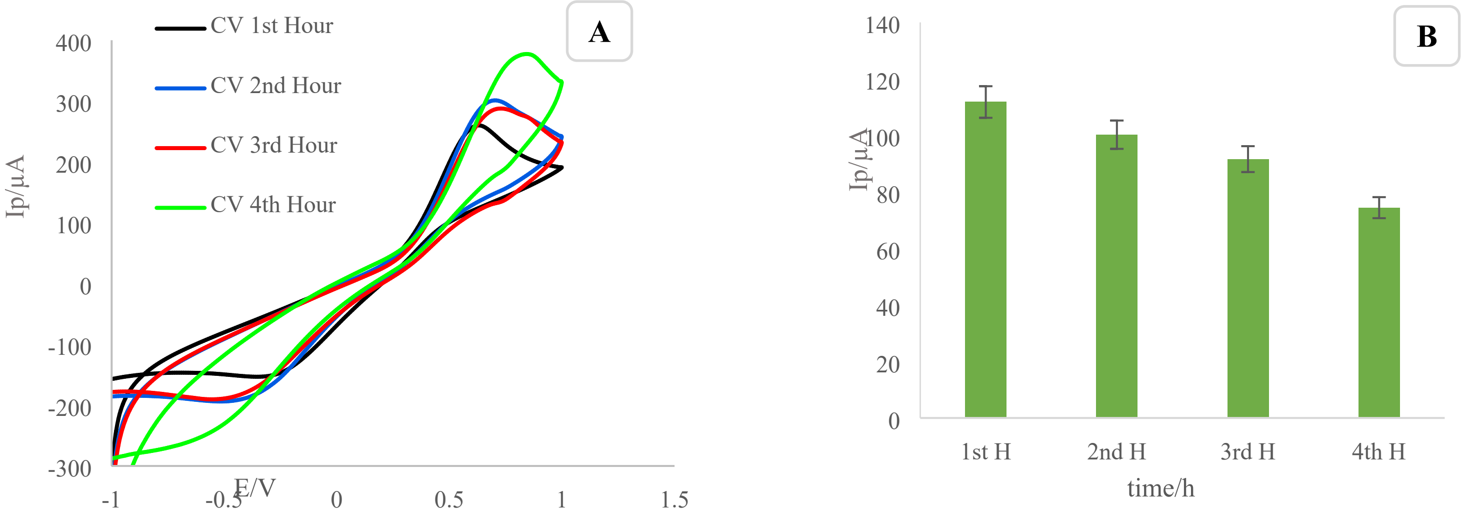

Fig.S3. A) CVs of ITO-PET-AuNPs (CTAB) in different times of storage (1st, 2nd, 3rd and 4th hours), in the presence of $K_4Fe(CN)_6$ /$K_3Fe(CN)_6$ 0.5M containing KCl. **B)** Histogram of peak current in different storage times (per hour).

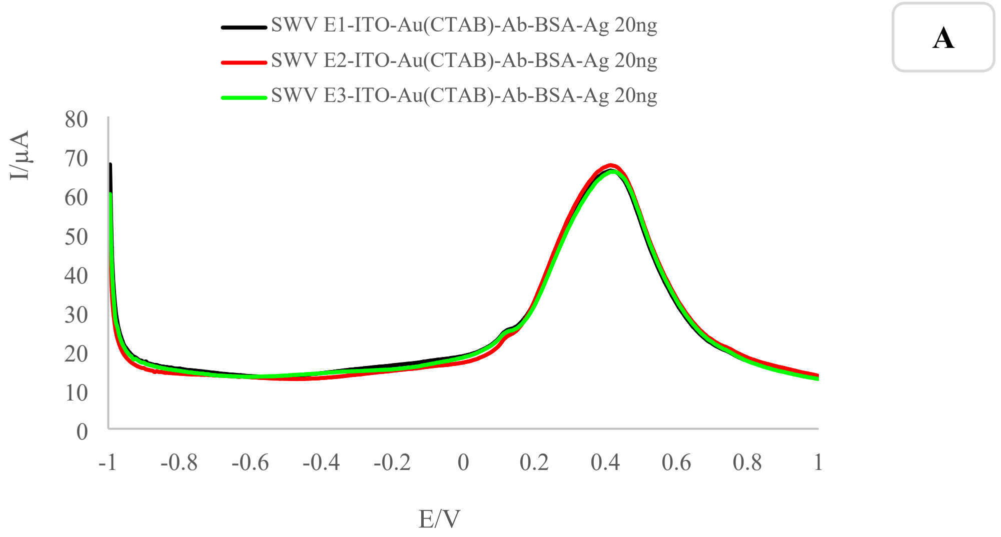

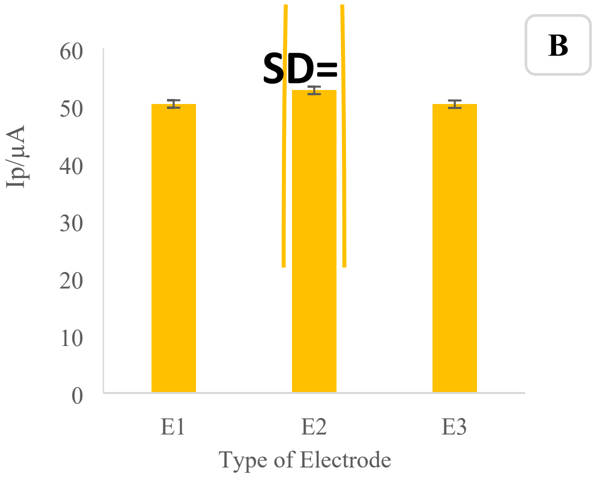
B
SD=
60
50
40
30
20
10
0
Ip/µA
E1
E2
E3
Type of Electrode

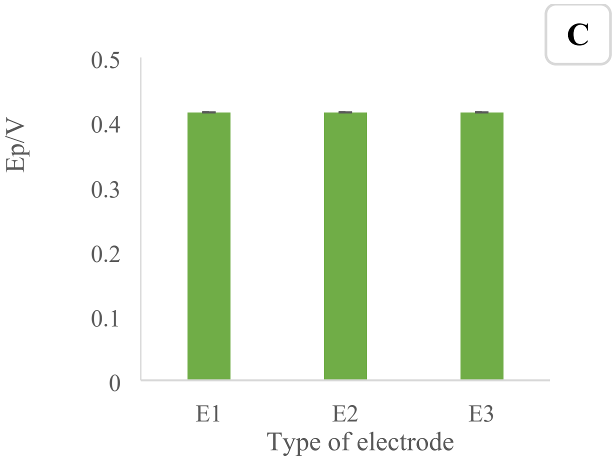
C
0.5
0.4
0.3
0.2
0.1
0
Ep/V
E1
E2
E3
Type of electrode

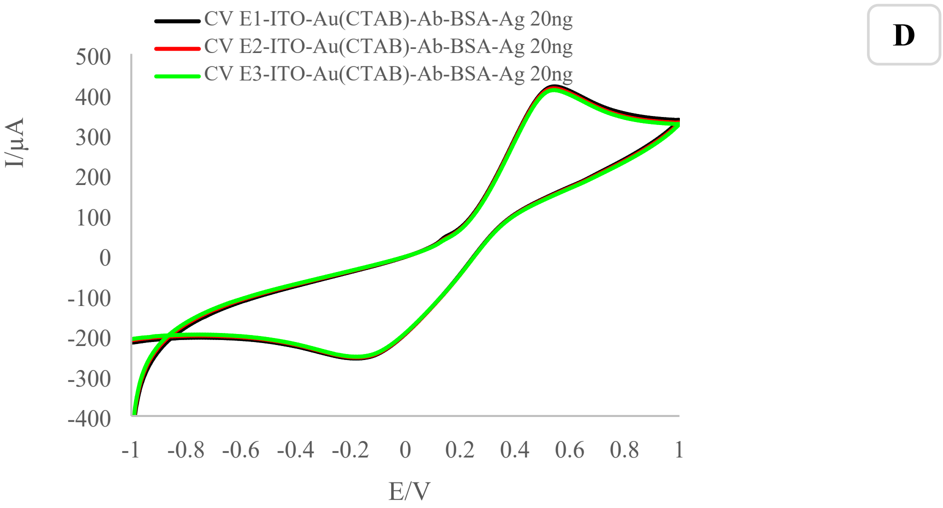
D
CV E1-ITO-Au(CTAB)-Ab-BSA-Ag 20ng
CV E2-ITO-Au(CTAB)-Ab-BSA-Ag 20ng
CV E3-ITO-Au(CTAB)-Ab-BSA-Ag 20ng
500
400
300
200
100
0
-100
-200
-300
-400
I/µA
-1
-0.8
-0.6
-0.4
-0.2
0
0.2
0.4
0.6
0.8
1
E/V

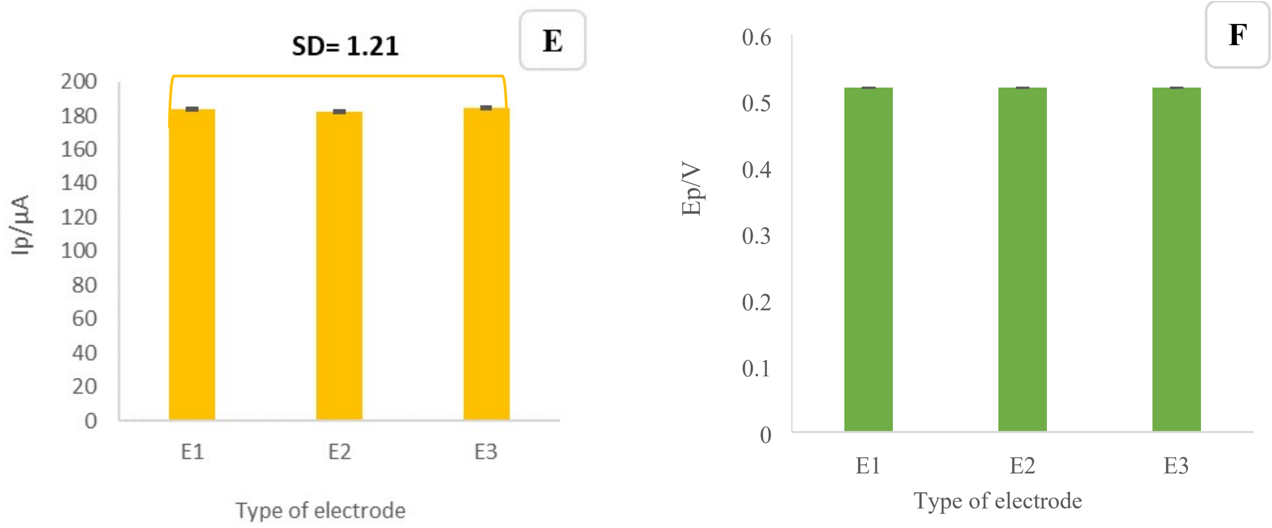

Fig. S4. A, D) SWV and CVs of engineered immunosensor reproducibility, in the presence of $K_4Fe(CN)_6$ /$K_3Fe(CN)_6$ 0.5M containing KCl (0.1 M). **B, C & E, F)** Histogram of peak current height (µA) and potential (V) of SWV and CV, respectively. **(RSD=** 0.0269%, 0.006%, **n=3**, respectively).

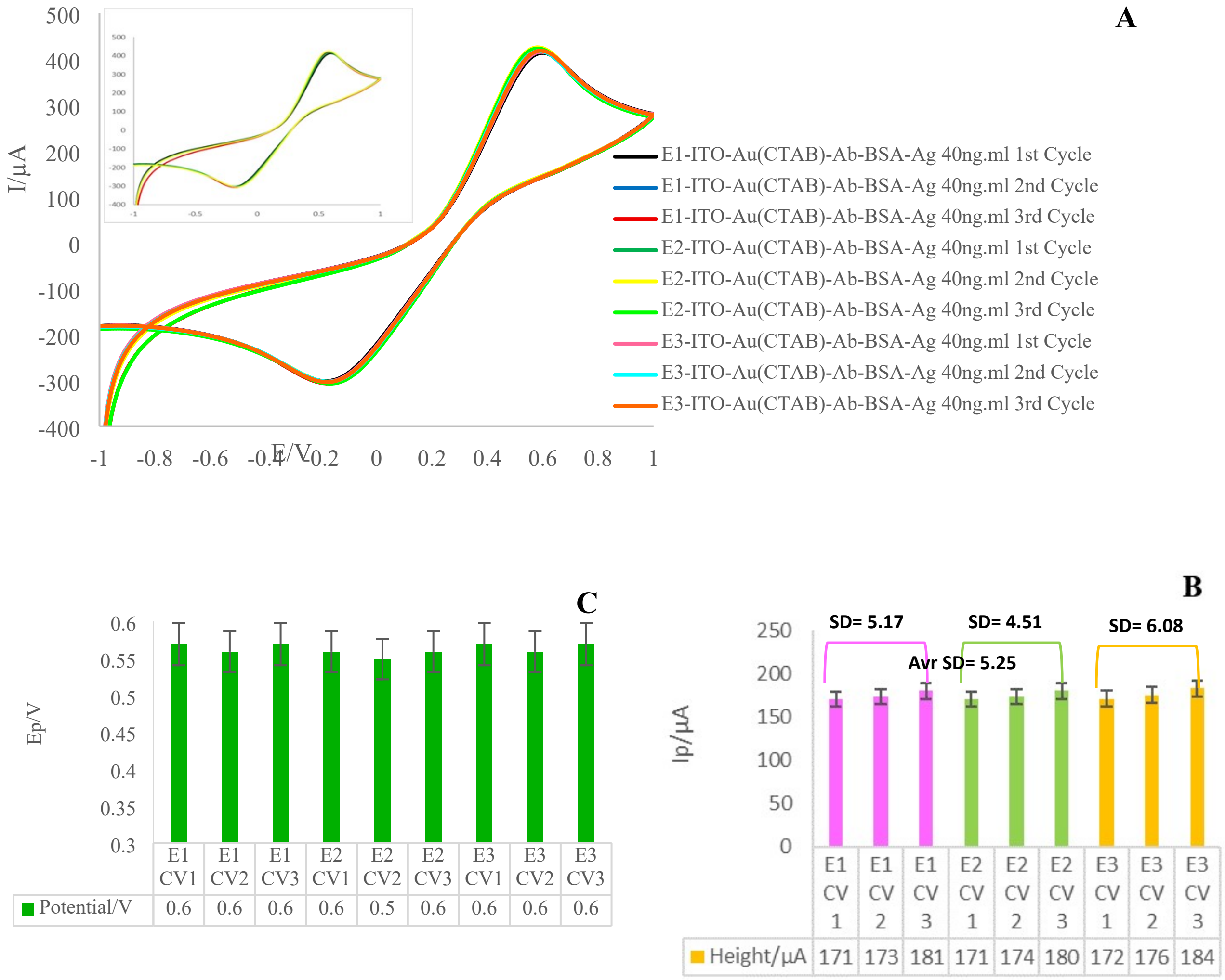

Fig. S5. A) CVs of engineered immunosensor repeatability, in the presence of $K_4Fe(CN)_6$ /$K_3Fe(CN)_6$ 0.5M containing KCl. Data information: ($T_{equilibration}$: 0 s, E_{begin}: -1.0 V, $E_{vertex1}$: 1.0 V, $E_{vertex2}$: -1.0 V, E_{step}: 0.01 V, scan rate: 0.1 V s^{-1}). **B &C)** Histogram of peak current (µA) and potential (V) of versus number of electrodes.

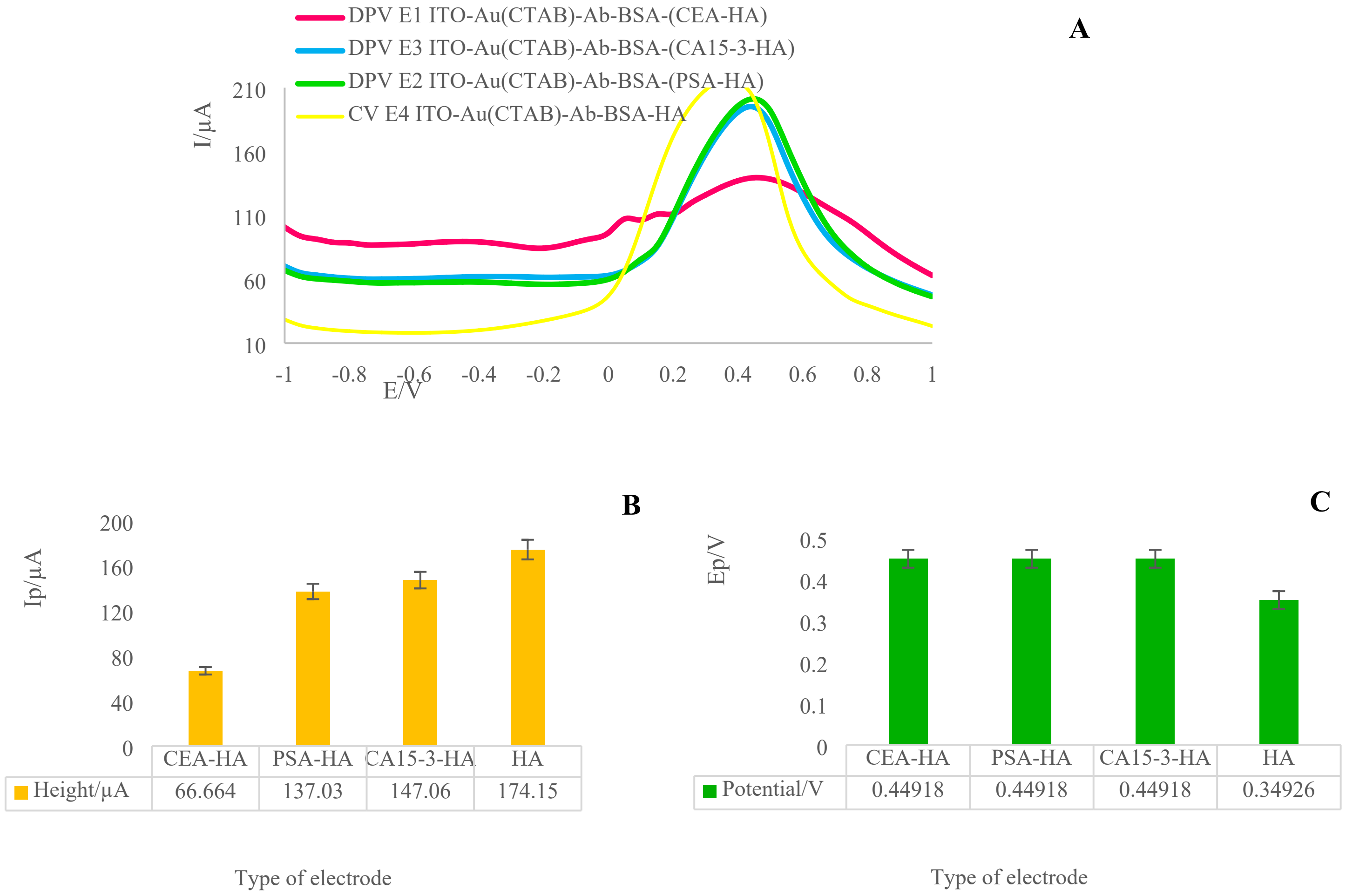

Fig. S6. A) DPVs of the fabricated immunosensor in the presence of HA and three interfering biomarkers, PSA (60 ng L^{-1}), CEA (75 ng L^{-1}) and CA15-3 (70 ng L^{-1}). DPV data information: ($T_{equilibration}$: 2 s, E_{begin}: -1.0 V, E_{end}: 1.0 V, E_{step}: 0.1 V, E_{pulse}: 0.005 V, T_{pulse}: 0.2 s, scan rate: 0.1 V s^{-1}), in the presence of $K_4Fe(CN)_6$ /$K_3Fe(CN)_6$ 0.5M containing KCl . **B & C)** Histogram of peak current and related potential (V) versus type of interfering agents.

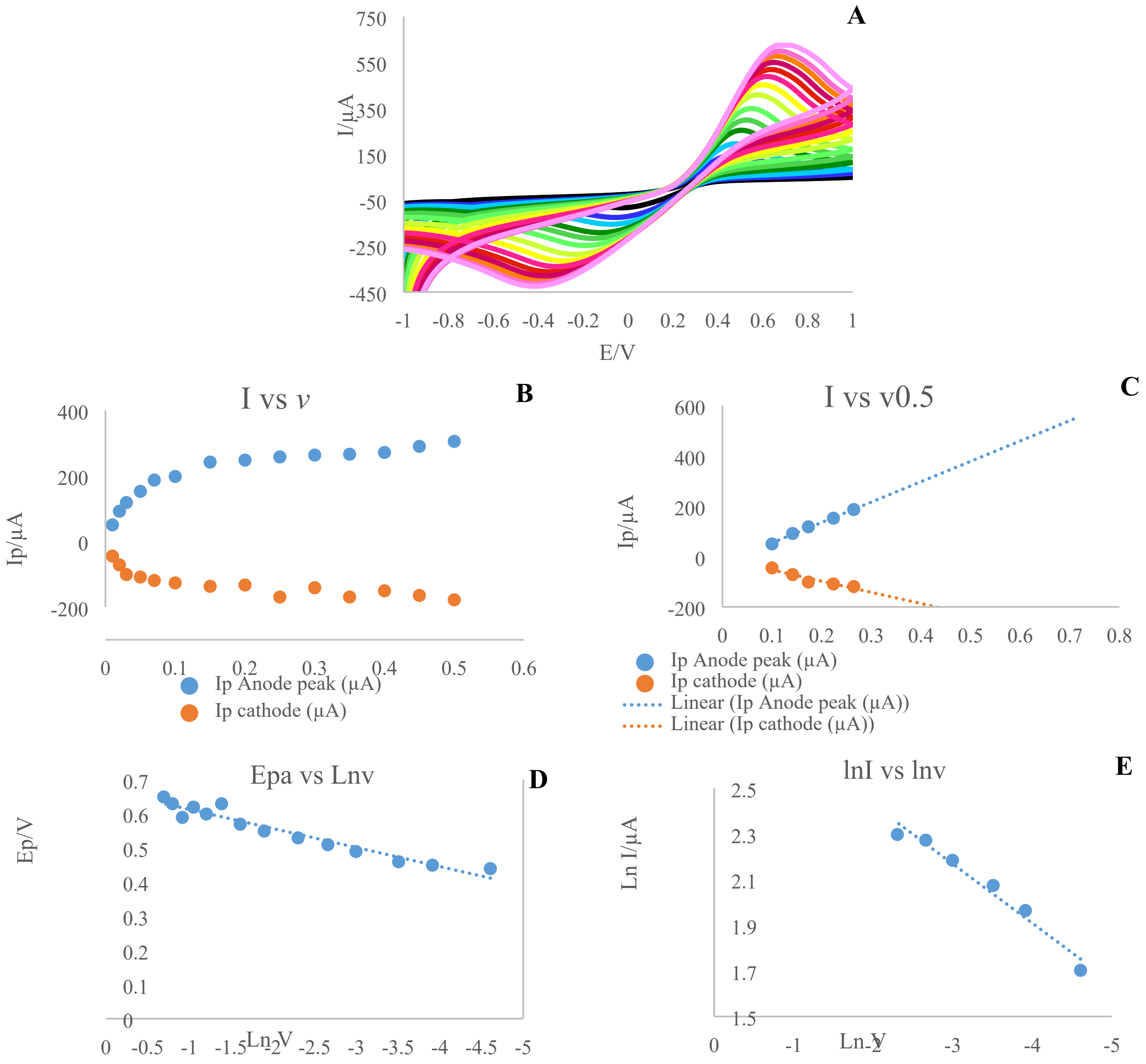

Fig. S7. A) CVs of AuNPs-CTAB in 0.01 M ($Fe(CN)_6^{3/4}$–KCl) in different potential sweep rates (from inner to outer): 10, 20, 30, 40, 50, 60, 70, 80, 90, 100, 200, 300, 400, 500, 600, 700, 800, 900, and 1000 mV s^{-1}. **B)** Plot of Ep versus log ν for cyclic voltammograms depicted in (A) for

anodic peaks (b1) and cathodic peaks (b2). **C-F)** The dependency of anodic **(C, E)** and cathodic **(D, F)** peak currents on the potential sweep rate at lower values of 2-75 mV s^{-1} **(C, E)** and on the square roots of sweep rate at higher values of 100-900 mV s^{-1} **(D, F).**
